# Accelerometer-derived physical activity and sedentary time and cardiac biomarkers: The Maastricht Study

**DOI:** 10.3389/fcvm.2023.1081713

**Published:** 2023-04-28

**Authors:** E. J. Vandercappellen, A. Koster, H. H. C. M. Savelberg, S. J. P. M. Eussen, P. C. Dagnelie, M. T. Schram, M. M. J. van Greevenbroek, A. Wesselius, J. P. Kooman, A. A. Kroon, R. M. A. Henry, C. D. A. Stehouwer

**Affiliations:** ^1^ Department of Internal Medicine, Maastricht University Medical Center+, Maastricht, The Netherlands; ^2^ CARIM School for Cardiovascular Diseases, Maastricht University, Maastricht, The Netherlands; ^3^ CAPHRI Care and Public Health Research Institute, Maastricht University, Maastricht, The Netherlands; ^4^ Department of Social Medicine, Maastricht University, Maastricht, The Netherlands; ^5^ Department of Nutrition and Movement Science, Maastricht University, Maastricht, The Netherlands; ^6^ NUTRIM School for Nutrition and Translational Research in Metabolism, Maastricht University, Maastricht, The Netherlands; ^7^ Department of Epidemiology, Maastricht University, Maastricht, The Netherlands; ^8^ Heart and Vascular Center, Maastricht University Medical Center+, Maastricht, The Netherlands

**Keywords:** physical activity, sedentary time, cardiac biomarkers, cardiac injury, type 2 diabetes

## Abstract

**Background:**

Cardiac troponins and NT-proBNP are biomarkers of cardiac injury that are used clinically in the diagnosis of myocardial infarction and heart failure. It is not known whether the amount, types and patterns of physical activity (PA) and sedentary behaviour are associated with levels of cardiac biomarkers.

**Methods:**

In the population-based Maastricht Study (*n* = 2,370, 51.3% male, 28.3% T2D) we determined cardiac biomarkers hs-cTnI, hs-cTnT, and NT-proBNP. PA and sedentary time were measured by activPAL and divided into quartiles [quartile 1 (Q1) served as reference]. The weekly pattern of moderate-to-vigorous PA (insufficiently active; regularly actives; weekend warriors) and coefficient of variation (CV) was calculated. Linear regression analyses were conducted with adjustment for demographic, lifestyle, and cardiovascular risk factors.

**Results:**

There was no consistent pattern between physical activity (different intensities: total, light, moderate-to-vigorous and vigorous) and sedentary time on the one hand and hs-cTnI and hs-cTnT on the other. Those with the highest levels of vigorous intensity PA had significantly lower levels of NT-proBNP. With regard to PA patterns, weekend warriors and regularly actives had lower levels of NT-proBNP but not with hs-cTnI and hs-cTnT (reference:insufficiently actives). A higher weekly moderate-to-vigorous PA CV (indicating more irregular activity) was associated with lower levels of hs-cTnI and higher levels of NT-proBNP, but not with hs-cTnT.

**Conclusions:**

In general, there was no consistent association between PA and sedentary time and cardiac troponins. In contrast, vigorous and possibly moderate-to-vigorous intensity PA, especially if done regularly, were associated with lower levels of NT-proBNP.

## 1. Introduction

Cardiac troponins and NT-proBNP are biomarkers of cardiac injury that are used clinically in the diagnosis of myocardial infarction and heart failure, respectively (1, 2). Additionally, several studies have shown that higher levels of these biomarkers, even outside the context of clinical disease, are associated with lower event-free survival (2–4) and higher cardiovascular morbidity and mortality (4, 5).

Physical activity is a well-known means to improve cardiovascular function and longevity (6). Nevertheless, in some people, acute bouts of exercise have been associated with higher levels of cardiac biomarkers (3, 7–9), although the prognostic significance of this observation is uncertain (3, 9–12). It is also not clear whether habitual physical activity or patterns of physical activity are associated with higher or lower levels of cardiac biomarkers. Two studies found no clear associations between moderate-to-vigorous physical activity, as measured with an accelerometer, and cardiac troponins or NT-proBNP (12, 13). Additionally, these studies observed that higher levels of sedentary behaviour were associated with higher levels of cardiac troponins (13) but not NT-proBNP (12). However, these previous studies did not assess physical activity in different intensities including light and vigorous physical activity in relation to cardiac biomarkers. Moreover, we do not know if the patterns of physical activity (e.g., the spread over the week) may be related to levels of cardiac biomarkers.

Individuals with type 2 diabetes have higher levels of cardiac biomarkers compared with individuals with normal glucose metabolism which, in general, are also associated with adverse cardiovascular outcomes (14–19). However, the association of intensity or patterns of physical activity with cardiac biomarkers in people with type 2 diabetes has not been investigated.

We hypothesised that physical activity is inversely associated with cardiac biomarkers and sedentary time is positively associated with cardiac biomarkers and if the association is stronger in individuals with type 2 diabetes. In the view of the above, we investigated the relationship between the amount of physical activity in different intensities, and the pattern of physical activity over the week and sedentary time on the one hand and cardiac biomarkers on the other in a large population-based cohort with an oversampling of type 2 diabetes.

## 2. Methods

### 2.1. Study population

We used data from the Maastricht Study, an observational prospective population-based cohort study. The rationale and methodology have been described previously (20).

In brief, the study focuses on the etiology, pathophysiology, complications, and comorbidities of type 2 diabetes and is characterized by an extensive phenotyping approach. Eligible participants were individuals between 40 and 75 years of age and living in the southern part of the Netherlands. The population consisted mainly of Caucasian participants (>98%). Participants were recruited through mass media campaigns and from the municipal registries and the regional Diabetes Patient Registry *via* mailings. Recruitment was stratified according to known type 2 diabetes status, with an oversampling of individuals with type 2 diabetes, for reasons of efficiency. This study included cross-sectional data from 3,451 participants, who completed the baseline survey between November 2010 and September 2013. The examinations of each participant were performed within a time window of three months.

The study was approved by the institutional medical ethical committee (NL31329.068.10) and the Minister of Health, Welfare, and Sports of the Netherlands (permit no. 131088-105234-PG). All participants gave written informed consent.

### 2.2. Measurements

#### 2.2.1. Cardiac biomarkers (outcomes)

Fasting blood samples were collected from all participants and processed according to the manufacturers' instructions. Serum samples were stored at −80°C for 1–4 years. High-sensitivity cardiac troponin T (hs-cTnT) was measured on a Roche Cobas 6000 analyzer (Roche) with the Elecsys Troponin T hs assay (Roche), which has a limit of blank (LoB) of 3 ng/L and a limit of detection (LoD) of 5 ng/L, and achieves a 10% coefficient of variation (10% CV) at 13 ng/L. High-sensitivity cardiac troponin I (hs-cTnI) was measured on an Architect i2000 SR analyzer (Abbott Diagnostics) with the Architect STAT High Sensitive Troponin-I assay (Abbott), which has an LoB range of 0.7–1.3 ng/L and an LoD range of 1.1–1.9 ng/L, and achieves a 10% CV at 4.7 ng/L. N-terminal pro-B-type natriuretic peptide (NT-proBNP) was assessed on a Roche Cobas 6000 analyzer (Roche) with the Elecsys proBNP II assay (Roche), which has a LoD of 5.0 ng/L (LoB not reported), and achieves a 20% CV at 50.0 ng/L. NT-proBNP and hs-cTNT concentrations below the LoD or LoB respectively, were set at LoD/2 and LoB/2, respectively (21).

#### 2.2.2. Physical activity, activity patterns and sedentary behavior (determinants)

Physical activity and sedentary time were measured using the activPAL3™ physical activity monitor (PAL Technologies, Glasgow, UK). The activPAL3 is a small (53 × 35 × 7 mm), lightweight (15 g) triaxial accelerometer that records movement in the vertical, anteroposterior and mediolateral axes, and also determines posture (sitting or lying, standing and stepping) based on acceleration information. The device was attached directly to the skin on the front of the right thigh with transparent 3M Tegaderm™ tape, after the device had been waterproofed using a nitrile sleeve. Participants were asked to wear the accelerometer for eight consecutive days, without removing it at any time. To avoid inaccurately identifying non-wear time, participants were asked not to replace the device once removed. Data were uploaded using the activPAL software and processed using customised software written in MATLAB R2018b (MathWorks, Natick, MA, USA) (22). Data from the first day were excluded from the analysis because participants performed physical function tests at the research centre after the device was attached. In addition, data from the final wear day providing ≤14 waking hours of data were excluded from the analysis. Participants were included if they provided at least 1 valid day (≥10 h of waking data) and at least six valid days for the weekly pattern.

We calculated the amount of time per week spent in light intensity physical activity (defined as standing and <100 steps/min), moderate-to-vigorous physical activity (defined as ≥100 steps/min), and vigorous intensity physical activity (defined as ≥130 steps/min) (23, 24). Total physical activity per day was defined as mean time spent stepping during waking time. Weekly activity pattern categories based on moderate-to-vigorous intensity physical activity were defined as: insufficiently active, <150 min moderate-to-vigorous intensity physical activity/week; and sufficiently active, ≥150 min moderate-to-vigorous intensity physical activity/week (based on international physical activity guidelines) (25). The sufficiently active category was further subdivided into “weekend warriors” and regularly actives. In accordance with previous research, weekend warriors were defined as participants who did ≥50% of the weekly moderate-to-vigorous intensity physical activity on only 1 or 2 days (26). Regularly actives were participants who did their moderate-to-vigorous intensity physical activity in ≥3 days. Thus, we defined three groups: (1) insufficiently active (0–150 min moderate-to-vigorous intensity physical activity/week); (2) weekend warriors (≥150 min moderate-to-vigorous intensity physical activity/week with more than 50% of the moderate-to-vigorous intensity physical activity in 1 or 2 days); and (3) regularly actives (≥150 min moderate-to-vigorous intensity physical activity/week in ≥3 days). Also, we assessed the variation of moderate-to-vigorous intensity physical activity/week per individual as a continuous variable by calculating the coefficient of variation (=SD/mean).

The total amount of sedentary time was based on the sedentary posture (sitting or lying), and calculated as the mean time spent in a sedentary position during waking time per day. The method used to determine waking time has been described elsewhere (22). In brief, an automated algorithm identified wake and bed times on an individual level on multiple days, i.e., different wake and bed times for each day for each participant. The algorithm is based on the number and duration of sedentary periods to identify bed times, and on the number and duration of active periods (standing or stepping) to identify wake times. The algorithm showed high accuracy in determining waking time compared with self-report, as the intra-class correlation coefficient (ICC) was 0.79 (*p* < 0.001) and the mean difference in waking time between both methods was 0.02 h (1.2 min), with limits of agreement of −1.1–1.2 h.

#### 2.2.3. Covariates

Covariates which were extracted from questionnaires included sex, age, level of education, smoking status, Dutch healthy diet index, mobility limitation, and history of cardiovascular disease. Level of education was categorized into low, medium, and high, and smoking status was categorized into never, former, and current smoker. Dietary habits were obtained from a validated food frequency questionnaire (27) and calculated as the adherence to Dutch healthy diet index (28). Mobility limitation was obtained from the 36-Item Short Form Health Survey questionnaire and was defined as having difficulty walking 500 m and/or climbing up a flight of stairs. Prevalent cardiovascular disease was defined as a self-reported history of myocardial infarction, cerebrovascular infarction or hemorrhage, or percutaneous artery angioplasty of, or vascular surgery on, the coronary, abdominal, peripheral, or carotid arteries. The use of lipid-modifying, antihypertensive and glucose-lowering medication was assessed during a medication interview (20). Body mass index (BMI), office blood pressure, ambulatory 24-hour blood pressure, triglycerides and total cholesterol-to-HDL ratio were determined as described elsewhere (20). Glucose metabolism status was assessed by medication use and using a 2 h oral glucose tolerance test and classified into normal glucose metabolism, prediabetes and type 2 diabetes according to the World Health Organization 2006 criteria (29).

Estimated glomerular filtration rate (eGFR) was estimated with the CKD-EPI equation based on the combination of serum creatinine and serum cystatin C. To assess urinary albumin excretion, participants were requested to collect two 24-h urine collections. UAE was preferably based on the average of two (available in 91.9% of the participants) 24-h urine collections (30).

### 2.3. Statistical analyses

All data were analysed using IBM SPSS software version 26.0 for Windows (IBM, Armonk, NY). Characteristics of the total study population and according to moderate-to-vigorous physical activity patterns were summarized as mean (SD), as percentages or as median [interquartile range] (in case of a skewed distribution). *p*-values were calculated by anova (continues) or *χ*^2^ test (categorical).

The cardiac biomarkers were positively skewed and log transformed for all analyses described below. Associations between physical activity (total, light, moderate-to-vigorous combined and vigorous separately), sedentary time and physical activity pattern (independent variables) and cardiac biomarkers (dependent variables) were examined with the use of multivariable linear regression models. Physical activity and sedentary time of the total population were divided into quartiles due to non-linear associations. These non-linear associations were also found with restricted cubic splines (STATA 17.0, Supplementary Figures S2–S4). Regression coefficients are presented as unstandardized coefficients. Models 1 and 2 were adjusted for potential confounders; models 3 and 4 were adjusted for variables that are potential confounders and/or mediators. Model 1 was adjusted for age, sex and glucose metabolism status; model 2 was additionally adjusted for smoking status, Dutch healthy diet index and level of education. Model 3 was additionally adjusted for history of cardiovascular disease, BMI, office systolic blood pressure, mobility limitations (yes/no), total cholesterol-to-HDL cholesterol ratio, triglycerides, lipid-modifying medication, anti-hypertensive medication, estimated glomerular filtration rate and albuminuria. For light intensity physical activity and sedentary time, model 4 was additionally adjusted for moderate-to-vigorous physical activity. For moderate-to-vigorous physical activity and vigorous physical activity, model 4 was additionally adjusted for sedentary time. The time out-of-bed can be defined as sedentary time, light intensity and moderate-to-vigorous physical activity. Therefore, it is conceivable that sedentary time is a confounder/mediator for moderate-to-vigorous and vigorous physical activity in the above analyses. Additionally, sedentary behavior and light intensity physical activity were further adjusted for moderate-to-vigorous physical activity, as a possible confounder/mediator, in the above analyses.

We performed interaction analyses for sex and glucose metabolism status in model 3. Next, we conducted several sensitivity analyses: we replaced BMI by waist circumference and replaced office blood pressure by 24-hour blood pressure. For all analyses, a *p*-value<0.05 was considered statistically significant.

## 3. Results

The study population for all analyses except those for activity patterns consisted of 2,370 participants. We excluded 806 with no accelerometer measurements, 18 with missing cardiac biomarkers and 257 who had missing data on confounders. Activity pattern analyses were done in 2022 participants, as 348 participants were additionally excluded because of accelerometer measurements of less than six days (Supplementary Figure S1). We compared the general characteristics of the included and excluded populations and found that most, but not all of the characteristics were similar (Supplementary Table S1).

Table 1 shows the characteristics of the total study population and according to the pattern of physical activity. The participants in the insufficiently active group, as compared to the more active participants, were older, more often male, more often current smokers, more often had mobility limitations, more often had type 2 diabetes, more often used medication, and had higher levels of cardiac biomarkers.

**Table 1:** Descriptive characteristics of the study population.

Characteristics	Total population (*n* = 2,370)	Phyiscal activity pattern (*n* = 2,022)			*p*-value
		Insufficiently actives (*n* = 175)	Weekend warriors (*n* = 497)	Regularly actives (*n* = 1,350)	
Socio-demographics					
Age (years)	61 (55; 66)	64 (59; 70)	63 (56; 67)	60 (54; 66)	<0.05
Sex (% male)	51.3	75.4	57.1	44.4	<0.05
Education level (%)					<0.05
• Low • Medium • High	33.7	50.3	33.2	31.4	
	28.1	29.1	24.1	28.6	
	38.2	20.6	42.7	40.0	
Lifestyle factors					
Smoking status (%)					<0.05
• Current • Former • Never	12.5	24.6	10.1	10.2	
	52.2	53.1	52.3	52.2	
	35.3	22.3	37.6	37.6	
Alcohol consumption (%)					<0.05
• None • Low • High	17.6	29.7	14.1	16.9	
	56.9	54.3	56.5	57.2	
	25.4	16.0	29.4	25.9	
Dutch healthy diet index	83.71 (14.67)	78.32 (14.79)	84.45 (13.74)	85.03 (14.71)	<0.05
Health-related factors					
Mobility limitations (%)	20.3	47.4	17.7	17.4	<0.05
BMI (kg/m^2^)	27.01 (4.51)	30.10 (5.46)	26.78 (3.99)	26.41 (4.09)	<0.05
History of CVD (%)	16.6	29.1	16.5	15.0	<0.05
Glucose metabolism status (%)					<0.05
• Normal • Impaired • Type 2 diabetes • Other type of diabetes	55.4	22.9	55.9	61.4	
	15.2	10.3	16.9	14.9	
	28.3	65.1	26.4	22.4	
	1.1	1.7	0.8	1.3	
Antihypertensive medication use (%)	41.6	69.1	42.7	38.0	<0.05
Lipid-modifying medication use (%)	37.9	64.6	37.8	33.1	<0.05
Glucose-lowering medication use (%)	23.7	58.3	21.3	18.6	<0.05
Total cholesterol-to-HDL cholesterol ratio	3.38 (2.76; 4.19)	3.63 (3.00; 4.60)	3.45 (2.82; 4.29)	3.28 (2.69; 4.06)	<0.05
Triglycerides (mmol/L)	1.22 (0.89; 1.73)	1.55 (1.11; 2.18)	1.25 (0.90; 1.76)	1.16 (0.86; 1.61)	<0.05
eGFR	88.98 (79.01; 98.44)	83.76 (71.52; 92.87)	88.50 (77.86; 97.46)	89.88 (79.79; 99.40)	<0.05
Albuminuria (%)	9.0	24.0	7.8	6.8	<0.05
Physical activity and sedentary time					
Total physical activity (min/day)	117.0 (89.4; 146.4)	60.0 (20.4; 72.6)	111.0 (89.4; 135.0)	125.4 (101.4; 153.0)	<0.05
Light intensity physical activity (min/day)	324.0 (91.2)	247.8 (82.8)	304.2 (78.6)	341.4 (86.4)	<0.05
Moderate-to-vigorous intensity physical activity (min/day)	51.0 (34.8; 69.6)	16.2 (11.4; 18.6)	50.4 (38.4; 50.4)	56.4 (40.8; 73.2)	<0.05
Vigorous intensity physical activity (min/day)	5.4 (2.4; 11.4)	1.2 (0.06; 1.8)	5.4 (3.00; 10.8)	6.0 (3.6; 12.6)	<0.05
Sedentary time (min/day)	565.2 (100.2)	658.2 (90.0)	585.0 (86.4)	544.8 (93.6)	<0.05
Cardiac biomarkers					
Hs-cTnI (ng/L)	1.90 (1.20; 3.10)	2.50 (1.60; 3.90)	2.00 (1.30; 3.30)	1.80 (1.20; 2.80)	<0.05
Hs-cTnT (ng/L)	5.50 (3.90; 8.04)	8.01 (5.14; 10.92)	5.57 (4.05; 8.13)	5.20 (3.64; 7.43)	<0.05
NT-proBNP (pmol/L)	6.09 (3.34; 10.88)	7.75 (3.89; 14.01)	6.22 (3.56; 10.47)	6.02 (3.30; 11.02)	<0.05

Values are means (SD) or median (Q1–Q3), unless stated otherwise. BMI, body mass index; eGFR, estimated glomerular filtration rate; Hs-cTnI, high-sensitivity cardiac troponins I; Hs-cTnT, high-sensitivity cardiac troponins T; NT-proBNP, N-terminal pro-B-type natriuretic peptide.

### 3.1. Hs-cTnI

In the fully adjusted model, individuals in quartile 2 of total physical activity had statistically significantly lower levels of hs-cTnI compared to the least active individuals in quartile 1 [unstandardized β (95% CI), Q2: −0.11 (−0.22; −0.01); Table 2, model 3]. For light intensity physical activity, individuals in quartiles 2 and 3 had significantly lower levels of hs-cTnI [Q2: −0.18 (−0.28; −0.08); Q3: −0.10 (−0.21; −0.002); Table 2, model 3] compared to participants in quartile 1. However, for vigorous intensity physical activity, individuals in quartile 4 had statistically significantly higher levels of hs-cTnI [Q4: 0.22 (0.11; 0.33); Table 2, model 3]. As for sedentary time, individuals in quartile 3 had statistically significantly lower levels of hs-cTnI [Q3: −0.12 (–0.22; −0.02); Table 2, model 3]. There was no association between moderate-to-vigorous intensity physical activity and hs-cTnI.

**Table 2:** Associations of physical activity and sedentary behavior with Hs-cTnI (categorical) (*n* = 2,370).

	Total physical activity			
Q1 (*n* = 592) (11.4–89.4 min/day)	Q2 (*n* = 593) (89.4–117.0 min/day)	Q3 (*n* = 593) (117.0–146.4 min/day)	Q4 (*n* = 592) (146.4–322.8 min/day)
Model 1	Ref	**−0.16** **(−0.26; −0.05)**	**−0.16** (**−0.27; −0.06)**	−0.09 (−0.20; 0.01)
Model 2	Ref	**−0.17** (**−0.27; −0.06)**	**−0.17** (**−0.28; −0.07)**	−0.10 (−0.21; 0.01)
Model 3	Ref	**−0.11** (**−0.22; −0.01)**	−0.08 (−0.19; 0.02)	0.01 (−0.09; 0.12)
	Light intensity physical activity			
	Q1 (*n* = 592) (46.2–259.2 min/day)	Q2 (*n* = 593) (259.2–319.8 min/day)	Q3 (*n* = 593) (319.8–385.8 min/day)	Q4 (*n* = 592) (385.8–734.4 min/day)
Model 1	Ref	**−0.21** (**−0.31; −0.10)**	**−0.15** (**−0.25; −0.04)**	**−0.11** (**−0.22; −0.001)**
Model 2	Ref	**−0.21** (**−0.31; −0.11)**	**−0.15** (**−0.26; −0.04)**	−0.10 (−0.21; 0.01)
Model 3	Ref	**−0.18** (**−0.28; −0.08)**	**−0.10** (**−0.21; −0.002)**	−0.03 (−0.13;0.08)
Model 4	Ref	**−0.19** (**−0.29; −0.09)**	**−0.12** (**−0.22; −0.02)**	−0.05 (−0.16;0.06)
	Moderate-to-vigorous intensity physical activity			
	Q1 (*n* = 592) (0.6–34.8 min/day)	Q2 (*n* = 593) (34.8–51.0 min/day)	Q3 (*n* = 593) (51.0–69.6 min/day)	Q4 (*n* = 592) (69.6–244.2 min/day)
Model 1	Ref	−0.05 (−0.16; 0.05)	−0.07 (−0.18; 0.03)	−0.07 (−0.18; 0.04)
Model 2	Ref	−0.06 (−0.17; 0.04)	−0.09 (−0.20; 0.02)	−0.09 (−0.19; 0.03)
Model 3	Ref	−0.02 (−0.13; 0.08)	−0.02 (−0.12; 0.09)	0.04 (−0.07; 0.15)
Model 4	Ref	−0.01 (−0.12; 0.09)	0.002 (−0.11; 0.11)	0.07 (−0.05; 0.18)
	Vigorous intensity physical activity			
	Q1 (*n* = 592) (0.0–2.4 min/day)	Q2 (*n* = 593) (2.4–5.4 min/day)	Q3 (*n* = 593) (5.4–11.4 min/day)	Q4 (*n* = 592) (11.4–107.4 min/day)
Model 1	Ref	−0.01 (−0.11; 0.10)	0.000 (−0.11; 0.11)	0.11 (−0.01; 0.21)
Model 2	Ref	−0.01 (−0.11; 0.10)	−0.01 (−0.12; 0.10)	0.10 (−0.01; 0.21)
Model 3	Ref	0.03 (−0.08; 0.13)	0.06 (−0.04; 0.17)	**0.22** (**0.11; 0.33)**
Model 4	Ref	0.04 (−0.06; 0.14)	0.08 (−0.03; 0.19)	**0.24** (**0.13; 0.35)**
	Sedentary time			
	Q1 (*n* = 592) (148.8–495.0 min/day)	Q2 (*n* = 593) (495.0–564.6 min/day)	Q3 (*n* = 593) (564.6–634.8 min/day)	Q4 (*n* = 592) (634.8–952.8 min/day)
Model 1	Ref	−0.02 (−0.13; 0.08)	−0.05 (−0.16; 0.05)	**0.17** (**0.06; 0.28)**
Model 2	Ref	−0.03 (−0.14; 0.07)	−0.06 (−0.17; 0.05)	**0.16** (**0.05; 0.27)**
Model 3	Ref	−0.07 (−0.17; 0.03)	**−0.12** (**−0.22; −0.02)**	0.06 (−0.05; 0.17)
Model 4	Ref	−0.05 (−0.15; 0.05)	−0.09 (−0.19; 0.02)	0.11 (−0.002; 0.23)

Regression results are presented as unstandardized coefficients (β), with 95% confidence intervals (95% CI). Boldface indicates statistical significance (*p* < 0.05). Model 1 was adjusted for age, sex, glucose metabolism status. Model 2 was additionally adjusted for smoking, Dutch healthy diet index and level of education. Model 3 was additionally adjusted for history of cardiovascular disease, BMI, mobility limitation (yes/no), triglycerides, total cholesterol-to-HDL cholesterol ratio, use of lipid-modifying medication, use of anti-hypertensive medication, office systolic blood pressure, estimated glomerular filtration rate and albuminuria. For sedentary behavior and light intensity physical activity, model 4 was additionally adjusted for moderate-to-vigorous physical activity. For moderate-to-vigorous physical activity and vigorous physical activity, model 4 was additionally adjusted for sedentary time.

### 3.2. Hs-cTnT

In the fully adjusted model, individuals in quartile 2 of total physical activity had statistically significantly lower levels of hs-cTnT compared to individuals in quartile 1 [Q2: −0.11 (−0.20; −0.02); Table 3, model 3]. For light intensity physical activity, individuals in quartile 4 had significantly lower levels of hs-cTnT [Q4: 0.16 (0.07; 0.25); Table 3, model 3] compared to participants in quartile 1. As for sedentary time, individuals in quartile 2 and 3 had statistically significantly lower levels of hs-cTnT [Q2: −0.13 (−0.22; −0.05); Q3: −0.17 (−0.26; −0.08); quartile 1 (lease sedentary) served as reference; Table 2, model 3]. There was no association between moderate-to-vigorous intensity physical activity and vigorous physical activity on the one hand and hs-cTnI on the other.

**Table 3:** Associations of physical activity (all intensities) and sedentary behavior with Hs-cTnT (categorical) (*n* = 2,370).

	Total physical activity			
Q1 (*n* = 592) (11.4–89.4 min/day)	Q2 (*n* = 593) (89.4–117.0 min/day)	Q3 (*n* = 593) (117.0–146.4 min/day)	Q4 (*n* = 592) (146.4–322.8 min/day)
Model 1	Ref	**−0.20** **(−0.29; −0.11)**	**−0.13** (**−0.22; −0.04)**	**−0.13** (**−0.22; −0.03)**
Model 2	Ref	**−0.19** (**−0.28; −0.09)**	**−0.12** (**−0.21; −0.03)**	**−0.11** (**−0.21; −0.02)**
Model 3	Ref	**−0.11** (**−0.20; −0.02)**	−0.001 (−0.09; 0.09)	0.02 (−0.07; 0.12)
	Light intensity physical activity			
	Q1 (*n* = 592) (46.2–259.2 min/day)	Q2 (*n* = 593) (259.2–319.8 min/day)	Q3 (*n* = 593) (319.8–385.8 min/day)	Q4 (*n* = 592) (385.8–734.4 min/day)
Model 1	Ref	**−0.10** (**−0.19; −0.01)**	−0.09 (−0.18; 0.01)	0.06 (−0.04; 0.15)
Model 2	Ref	−0.09 (−0.18; 0.002)	−0.08 (−0.17; 0.01)	0.07 (−0.03; 0.16)
Model 3	Ref	−0.06 (−0.14; 0.03)	−0.01 (−0.10; 0.08)	**0.16** (**0.07; 0.25)**
Model 4	Ref	−0.05 (−0.14; 0.03)	−0.01 (−0.10; 0.08)	**0.16** (**0.07; 0.25)**
	Moderate-to-vigorous intensity physical activity			
	Q1 (*n* = 592) (0.6–34.8 min/day)	Q2 (*n* = 593) (34.8–51.0 min/day)	Q3 (*n* = 593) (51.0–69.6 min/day)	Q4 (*n* = 592) (69.6–244.2 min/day)
Model 1	Ref	**−0.12** (**−0.21; −0.03)**	**−0.13** (**−0.23; −0.04)**	**−0.15** (**−0.24; −0.06)**
Model 2	Ref	**−0.12** (**−0.21; −0.02)**	**−0.13** (**−0.22; −0.03)**	**−0.14** (**−0.24; −0.05)**
Model 3	Ref	−0.06 (−0.15; 0.03)	−0.04 (−0.13; 0.06)	−0.01 (−0.10; 0.09)
Model 4	Ref	−0.07 (−0.16; 0.02)	−0.05 (−0.15; 0.04)	−0.03 (−0.12; 0.07)
	Vigorous intensity physical activity			
	Q1 (*n* = 592) (0.0–2.4 min/day)	Q2 (*n* = 593) (2.4–5.4 min/day)	Q3 (*n* = 593) (5.4–11.4 min/day)	Q4 (*n* = 592) (11.4–107.4 min/day)
Model 1	Ref	0.01 (−0.09; 0.10)	−0.08 (−0.17; 0.01)	−0.04 (−0.14; 0.05)
Model 2	Ref	0.02 (−0.08; 0.11)	−0.07 (−0.17; 0.02)	−0.03 (−0.13; 0.07)
Model 3	Ref	0.06 (−0.03; 0.14)	−0.01 (−0.10; 0.08)	0.09 (0.000; 0.19)
Model 4	Ref	0.05 (−0.04; 0.14)	−0.02 (−0.11; 0.08)	0.09 (−0.01; 0.18)
	Sedentary time			
	Q1 (*n* = 592) (148.8–495.0 min/day)	Q2 (*n* = 593) (495.0–564.6 min/day)	Q3 (*n* = 593) (564.6–634.8 min/day)	Q4 (*n* = 592) (634.8–952.8 min/day)
Model 1	Ref	**−0.09** (**−0.18; −0.001)**	**−0.10** (**−0.19; −0.01)**	0.05 (−0.05; 0.14)
Model 2	Ref	**−0.10** (**−0.18; −0.01)**	**−0.11** (**−0.20; −0.02)**	0.03 (−0.06; 0.13)
Model 3	Ref	**−0.13** (**−0.22; −0.05)**	**−0.17** (**−0.26; −0.08)**	−0.07 (−0.16; 0.02)
Model 4	Ref	**−0.13** (**−0.22; −0.05)**	**−0.17** (**−0.26; −0.08)**	−0.07 (−0.17; 0.03)

Regression results are presented as unstandardized coefficients (β), with 95% confidence intervals (95% CI). Boldface indicates statistical significance (*p* < 0.05). Model 1 was adjusted for age, sex, glucose metabolism status. Model 2 was additionally adjusted for smoking, Dutch healthy diet index and level of education. Model 3 was additionally adjusted for history of cardiovascular disease, BMI, mobility limitation (yes/no), triglycerides, total cholesterol-to-HDL cholesterol ratio, use of lipid-modifying medication, use of anti-hypertensive medication, office systolic blood pressure, estimated glomerular filtration rate and albuminuria. For the sedentary behavior and light intensity physical activity, model 4 was additionally adjusted for moderate-to-vigorous physical activity. For moderate-to-vigorous physical activity and vigorous physical activity, model 4 was additionally adjusted for sedentary time.

### 3.3. NT-proBNP

In the fully adjusted model, individuals in quartile 4 of vigorous intensity physical activity had statistically significantly lower levels of NT-proBNP compared to individuals in quartile 1 [Q4: −0.12 (−0.23; −0.01); Table 4, model 3]. There was no association between total, light, moderate-to-vigorous intensity physical activity or sedentary time on the one hand and NT-proBNP on the other [quartile 1 served as reference; Table 4, model 3].

**Table 4:** Associations of physical activity and sedentary behavior with NT-proBNP (categorical) (*n* = 2,370).

	Total physical activity			
Q1 (*n* = 592) (11.4–89.4 min/day)	Q2 (*n* = 593) (89.4–117.0 min/day)	Q3 (*n* = 593) (117.0–146.4 min/day)	Q4 (*n* = 592) (146.4–322.8 min/day)
Model 1	Ref	−0.08 (−0.19; 0.03)	**−0.12** **(−0.23; −0.01)**	**−0.16** (**−0.27; −0.05)**
Model 2	Ref	−0.07 (−0.18; 0.04)	−0.10 (−0.21; 0.01)	**−0.14** (**−0.25; −0.02)**
Model 3	Ref	−0.05 (−0.16; 0.05)	−0.04 (−0.15; 0.07)	−0.08 (−0.20; 0.03)
	Light intensity physical activity			
	Q1 (*n* = 592) (46.2–259.2 min/day)	Q2 (*n* = 593) (259.2–319.8 min/day)	Q3 (*n* = 593) (319.8–385.8 min/day)	Q4 (*n* = 592) (385.8–734.4 min/day)
Model 1	Ref	−0.01 (−0.12; 0.10)	−0.02 (−0.13; 0.09)	−0.03 (−0.14; 0.08)
Model 2	Ref	−0.001 (−0.11; 0.11)	−0.001 (−0.11; 0.11)	−0.02 (−0.13; 0.10)
Model 3	Ref	0.01 (−0.09; 0.12)	0.03 (−0.08; 0.14)	0.01 (−0.10; 0.12)
Model 4	Ref	0.02 (−0.08; 0.13)	0.04 (−0.06; 0.15)	0.03 (−0.08; 0.14)
	Moderate-to-vigorous intensity physical activity			
	Q1 (*n* = 592) (0.6–34.8 min/day)	Q2 (*n* = 593) (34.8–51.0 min/day)	Q3 (*n* = 593) (51.0–69.6 min/day)	Q4 (*n* = 592) (69.6–244.2 min/day)
Model 1	Ref	−0.02 (−0.13; 0.09)	−0.03 (−0.14; 0.08)	**−0.17** (**−0.28; −0.05)**
Model 2	Ref	−0.01 (−0.12; 0.10)	−0.02 (−0.13; 0.09)	**−0.14** (**−0.26; −0.03)**
Model 3	Ref	0.003 (−0.10; 0.11)	0.03 (−0.08; 0.13)	−0.08 (−0.19; 0.03)
Model 4	Ref	−9.77^E^−5 (−0.11; 0.11)	0.02 (−0.09; 0.13)	−0.09 (−0.21; 0.03)
	Vigorous intensity physical activity			
	Q1 (*n* = 592) (0.0–2.4 min/day)	Q2 (*n* = 593) (2.4–5.4 min/day)	Q3 (*n* = 593) (5.4–11.4 min/day)	Q4 (*n* = 592) (11.4–107.4 min/day)
Model 1	Ref	−0.07 (−0.17; 0.04)	−0.06 (−0.17; 0.06)	**−0.19** (**−0.30; −0.08)**
Model 2	Ref	−0.05 (−0.16; 0.06)	−0.05 (−0.16; 0.06)	**−0.17** (**−0.29; −0.06)**
Model 3	Ref	−0.04 (−0.14; 0.07)	−0.03 (−0.14; 0.08)	**−0.12** (**−0.23; −0.01)**
Model 4	Ref	−0.04 (−0.15; 0.07)	−0.04 (−0.15; 0.07)	**−0.12** (**−0.24; −0.01)**
	Sedentary time			
	Q1 (*n* = 592) (148.8–495.0 min/day)	Q2 (*n* = 593) (495.0–564.6 min/day)	Q3 (*n* = 593) (564.6–634.8 min/day)	Q4 (*n* = 592) (634.8**–**952.8 min/day)
Model 1	Ref	0.08 (−0.03; 0.19)	−0.02 (−0.13; 0.09)	0.07 (−0.05; 0.18)
Model 2	Ref	0.08 (−0.03; 0.19)	−0.03 (−0.14; 0.08)	0.05 (−0.06; 0.17)
Model 3	Ref	0.07 (−0.04; 0.17)	−0.05 (−0.16; 0.05)	0.02 (−0.10; 0.13)
Model 4	Ref	0.05 (−0.05; 0.15)	−0.08 (−0.19; 0.03)	−0.03 (−0.15; 0.09)

Regression results are presented as unstandardized coefficients (β), with 95% confidence intervals (95% CI). Boldface indicates statistical significance (*p* < 0.05). Model 1 was adjusted for age, sex, glucose metabolism status. Model 2 was additionally adjusted for smoking, Dutch healthy diet index and level of education. Model 3 was additionally adjusted for history of cardiovascular disease, BMI, mobility limitation (yes/no), triglycerides, total cholesterol-to-HDL cholesterol ratio, use of lipid-modifying medication, use of anti-hypertensive medication, office systolic blood pressure, estimated glomerular filtration rate and albuminuria. For sedentary behavior and Light intensity physical activity, model 4 was additionally adjusted for moderate-to-vigorous physical activity. For moderate-to-vigorous physical activity and vigorous physical activity, model 4 was additionally adjusted for sedentary time.

### 3.4. Additional analyses

To investigate the mutual independence of the above associations, we adjusted light intensity physical activity and sedentary time for moderate-to-vigorous physical activity, and moderate-to-vigorous and vigorous physical activity for sedentary time. In general, the results were consistent with previous results (Tables 2–4, model 4).

After adjustment for demographic, lifestyle and cardiovascular risk factors, the physical activity patterns weekend warriors and regularly actives were inversely associated with NT-proBNP. There was no association between the physical activity pattern and hs-cTnI and hs-cTnT. For the coefficient of variation, there was an inverse association with hs-cTnI and a positive association with NT-proBNP, but not with hs-cTnT (Table 5, model 3).

**Table 5:** Associations of physical activity pattern and coefficient of variation (CV) and cardiac biomarkers.

	Insufficiently actives	Weekend Warriors	Regularly actives	CV
	Hs-cTnI			
Model 1	Ref	−0.03 (−0.19; 0.13)	−0.12 (−0.27; 0.03)	−0.01 (−0.02; 0.004)
Model 2	Ref	−0.06 (−0.22; 0.10)	−0.14 (−0.30; 0.01)	−0.01 (−0.02; 0.01)
Model 3	Ref	0.01 (−0.15; 0.17)	−0.06 (−0.21; 0.09)	**−0.01** **(−0.02; −0.003)**
	Hs-cTnT			
Model 1	Ref	**−0.16** **(−0.30; −0.02)**	**−0.17** **(−0.30; −0.04)**	0.002 (−0.01; 0.01)
Model 2	Ref	**−0.15** **(−0.29; −0.01)**	**−0.16** **(−0.29; −0.03)**	0.001 (−0.01; 0.01)
Model 3	Ref	−0.06 (−0.20; 0.08)	−0.06 (−0.19; 0.07)	−0.01 (−0.01; 0.002)
	NT-proBNP			
Model 1	Ref	**−0.27** **(−0.44; −0.11)**	**−0.37** **(−0.46; −0.15)**	**0.02** **(0.002; 0.03)**
Model 2	Ref	**−0.25** **(−0.42; −0.09)**	**−0.29** **(−0.44; −0.13)**	**0.01** **(0.000; 0.03)**
Model 3	Ref	**−0.21** **(−0.37; −0.04)**	**−0.25** **(−0.40; −0.10)**	**0.01** **(0.000; 0.03)**

Regression results are presented as unstandardized coefficients (β), with 95% confidence intervals (95% CI). Boldface indicates statistical significance (*p* < 0.05). Model 1 was adjusted for age, sex, glucose metabolism status. Model 2 was additionally adjusted for smoking, Dutch healthy diet index and level of education. Model 3 was additionally adjusted for history of cardiovascular disease, BMI, mobility limitation (yes/no), triglycerides, total cholesterol-to-HDL cholesterol ratio, use of lipid-modifying medication, use of anti-hypertensive medication, office systolic blood pressure, estimated glomerular filtration rate and albuminuria.

Interactions were tested in the fully adjusted model (model 3). There was no consistent statistically significant interaction with glucose metabolism status and sex (data not shown).

To test the robustness of the above results, we did several sensitivity analyses. In general, results remained similar when we replaced BMI by waist circumference (Supplementary Tables S2–S4) and when we replaced office systolic blood pressure with 24-hour blood pressure (Supplementary Tables S5–S7).

## 4. Discussion

This cross-sectional population-based study on the associations between accelerometer-measured physical activity and sedentary time on the one hand and cardiac biomarkers on the other had three main findings. First, there were no consistent associations between amounts or patterns of physical activity or sedentary time and levels of cardiac troponins. Second, the highest, as compared to the lowest quartile of vigorous and possibly moderate-to-vigorous physical activity was associated with lower levels of NT-proBNP, as was the pattern of moderate-to-vigorous physical activity: weekend warriors and regularly actives had significantly lower levels of NT-proBNP compared to the insufficiently actives. Additionally, there was an association between the CV and NT-proBNP, implying that less regularity of moderate-to-vigorous physical activity during the week was associated with higher levels of NT-proBNP. Third, associations in people with type 2 diabetes were similar to those in people without type 2 diabetes.

It is well-known that acute bouts of physical activity can induce cardiac troponin release (31–34). However, in our study, we did not find a consistent association between the different levels and patterns of physical activity and sedentary time on the one hand and cardiac troponins on the other. The underlying mechanism of the troponin release after an acute bout of exercise is not completely understood, but possible causes of troponin release after exercise are reversible injury (cell wounds, extracellular blebs, exocytosis), increased cardiomyocyte turnover, apoptosis and myocardial necrosis (3, 35, 36). Reassuringly, habitual physical activity or patterns of physical activity and sedentary time, as in our study, were not associated with levels of cardiac troponins, which is consistent which previous work on moderate-to-vigorous physical activity (12, 13), and with some (11) but not all (12) studies on sedentary behaviour.

In contrast, the highest quartile of vigorous (Q4 corresponds to >11.4 min/day) and possibly moderate-to-vigorous intensity physical activity was associated with lower levels of NT-proBNP. Earlier research reported an association between moderate physical activity and NT-proBNP (12, 37); our results suggests that such physical activity needs to be of at least moderate-to-vigorous intensity. This conclusion is further supported by the observation that weekend warriors and regularly actives had lower NT-proBNP levels than the insufficiently actives. Additionally, this study is the first to observe that the CV of moderate-to-vigorous activity was positively and significantly associated with NT-proBNP levels, implying that regularity of moderate-to-vigorous activity is beneficial with regard to this biomarker.

People with type 2 diabetes, as compared to those without, usually have higher levels of biomarkers of cardiac injury (14–19), which could be accompanied by associations of such biomarkers with physical activity that are weaker in people with compared to those without type 2 diabetes. Associations, however, were of similar strength, suggesting that benefits of physical activity in terms of cardiac biomarkers may be similar in people with and without type 2 diabetes.

This study had several strengths. We studied a large population-based sample with an oversampling of type 2 diabetes, so that we could study whether there was a difference between people with and without type 2 diabetes. Extensive measurements of physical activity were included (amount, intensity and pattern) measured by a thigh-worn accelerometer. We used multiple cardiac biomarkers that were measured by appropriate techniques. We also adjusted extensively for accurately measured possible confounders, which makes unmeasured and residual confounding less likely. The study also had several limitations. For the calculation of physical activity pattern, week and weekend days were not weighted. Causal inference should be made with great caution due to the cross-sectional design. Additionally, the population consisted mainly of Caucasian participants, which limits generalisability of our findings. Due to statistical reasons (non-linearity), we categorized physical activity and sedentary time into quartiles, which entails loss of information compared to analyses that use all (continuous) data. Finally, unmeasured or residual confounding cannot be definitively excluded.

In conclusion, our study shows that, independently of demographic, lifestyle and cardiovascular risk factors, in general there is no association between physical activity and sedentary time and cardiac troponins. In contrast, vigorous (>11 min/day) and possibly moderate-to-vigorous (>69 min/day) intensity physical activity, especially if done regularly, is associated with lower levels of NT-proBNP.

## Data Availability

The data sets generated during and/or analysed during the current study are not publicly available but are available from the corresponding author on reasonable request.
