# Supplementary material for: Accelerometer-derived physical activity and sedentary time and cardiac biomarkers: The Maastricht Study
Source: Front Cardiovasc Med. 2023 Apr 28;10:1081713. doi: 10.3389/fcvm.2023.1081713 (PMC10175613; doi:10.3389/fcvm.2023.1081713)
Supplement: Supplementary file 1 [file Table1.pdf]

Supplementary Material

1 Supplementary Figures and Tables

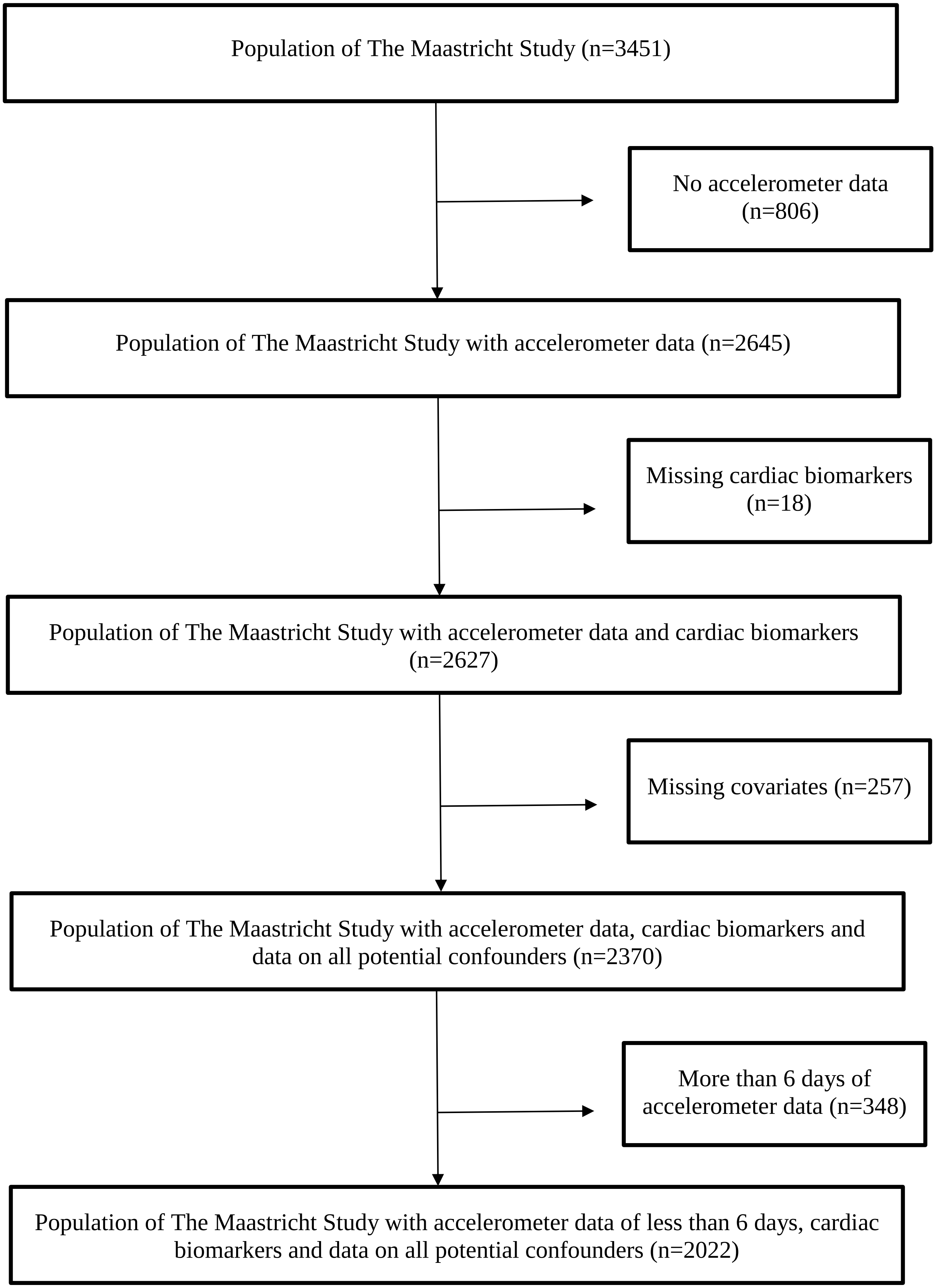
Population of The Maastricht Study (n=3451)
No accelerometer data (n=806)
Population of The Maastricht Study with accelerometer data (n=2645)
Missing cardiac biomarkers (n=18)
Population of The Maastricht Study with accelerometer data and cardiac biomarkers (n=2627)
Missing covariates (n=257)
Population of The Maastricht Study with accelerometer data, cardiac biomarkers and data on all potential confounders (n=2370)
More than 6 days of accelerometer data (n=348)
Population of The Maastricht Study with accelerometer data of less than 6 days, cardiac biomarkers and data on all potential confounders (n=2022)

Supplementary Figure 1. Selection of participants from The Maastricht Study cohort.

Supplementary Figure 2. Restricted cubic spline: hs-cTnI

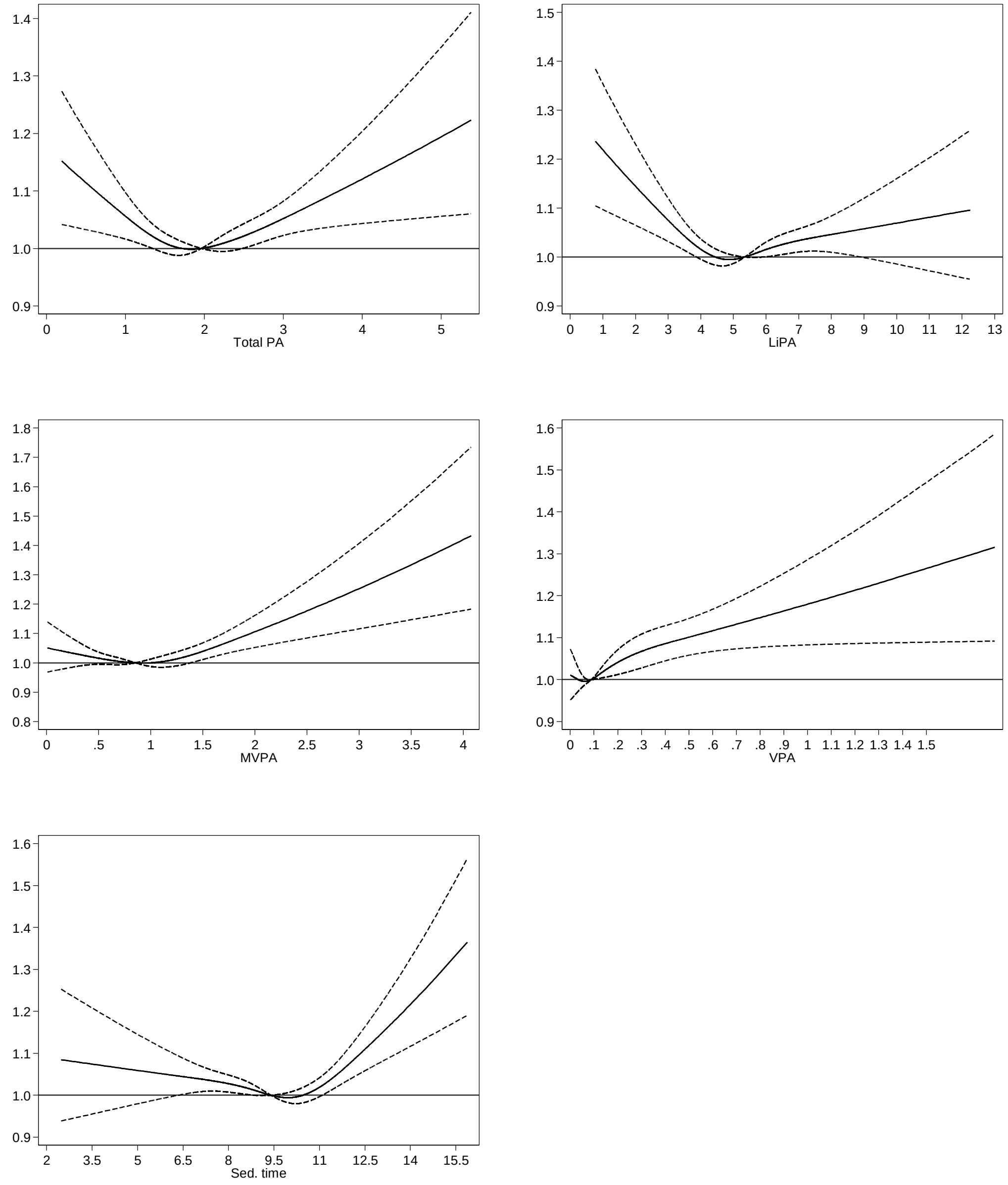

Supplementary Figure 3. Restricted cubic spline: hs-cTnT

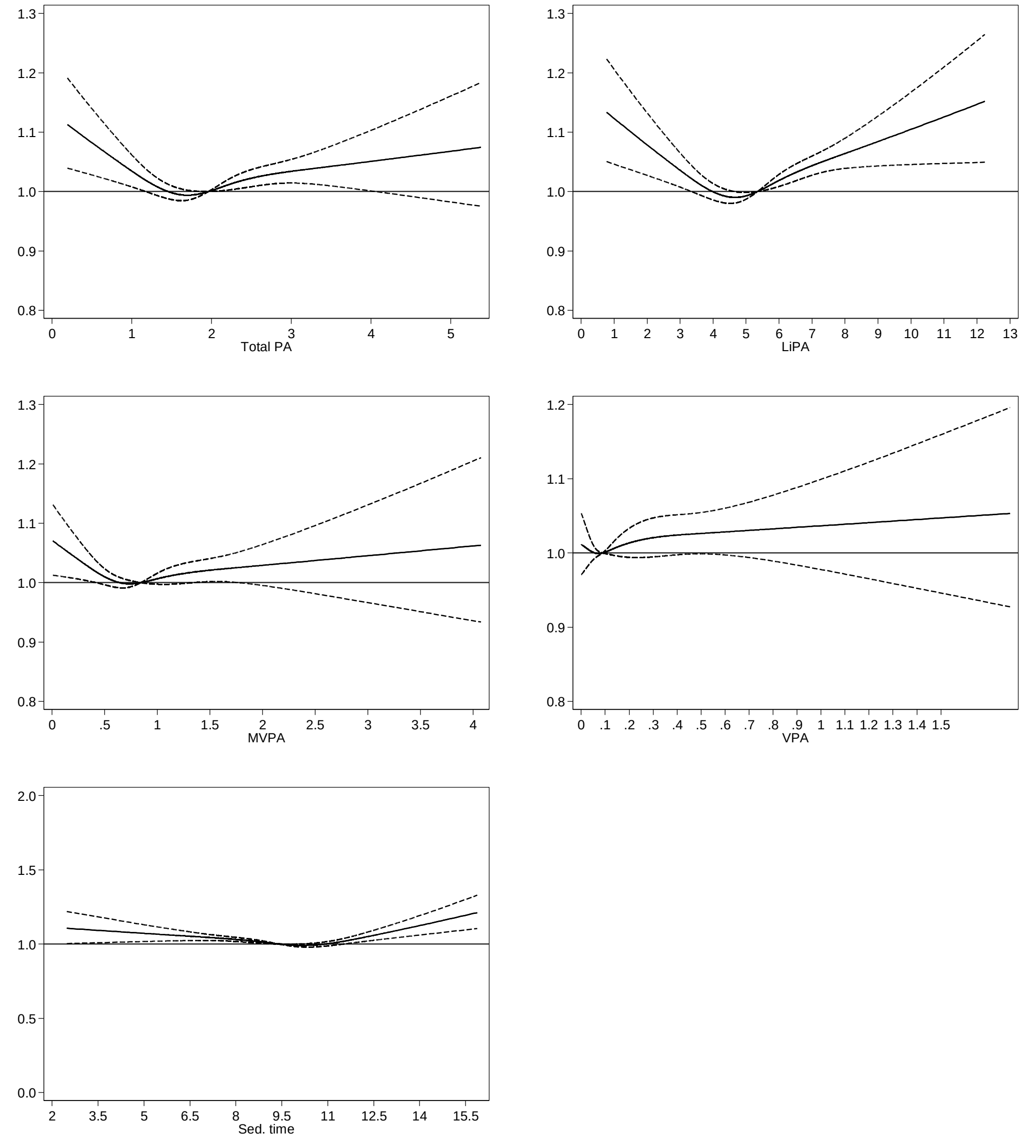

Supplementary Figure 4. Restricted cubic spline: NT-proBNP

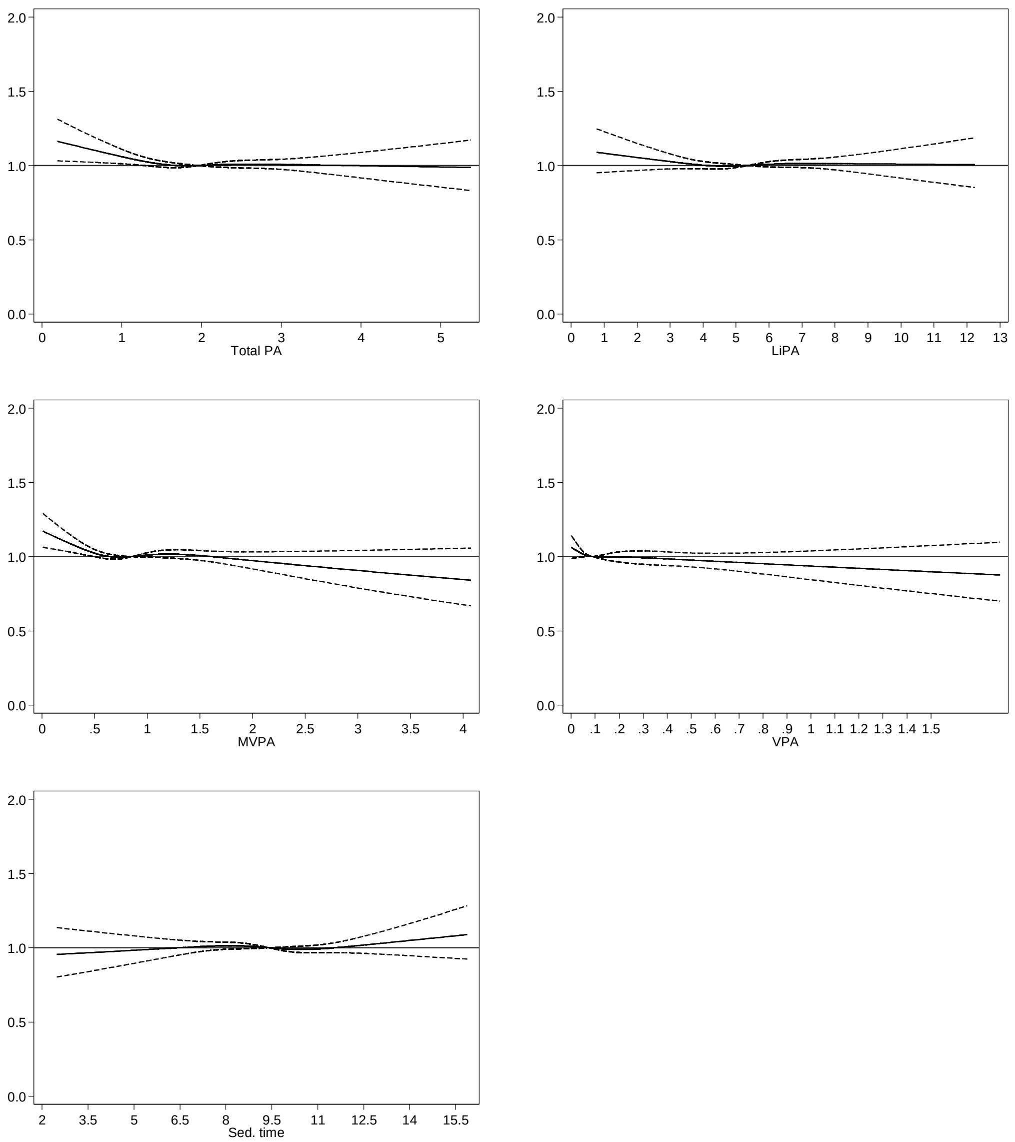

Supplementary table 1. in- and excluded participants

Characteristics	Included population (n=2370)	Excluded population (n=1081)	P-value
Age (years)	61 [55;66]	60 [53;66]	<0.01
Sex (% male)	51.5	51.7	>0.05
Education level (%)<br>• Low<br>• Medium<br>• High	 33.7<br>28.2<br>38.1	 33.1<br>28.6<br>38.0	>0.05
Smoking status (%)<br>• Current<br>• Former<br>• Never	 12.4<br>52.3<br>35.3	 16.9<br>50.5<br>32.6	<0.01
Alcohol consumption (%)<br>• None<br>• Low<br>• High	 17.6<br>56.9<br>25.4	 21.2<br>52.0<br>26.7	0.01
Mobility limitations (%)	20.3	25.6	<0.01
BMI (kg/m^2)	27.01 (4.51)	27.26 (4.67)	>0.05
History of CVD (%)	16.6	17.3	>0.05
Glucose metabolism status (%)<br>• Normal<br>• Impaired<br>• Type 2 diabetes<br>• Other type of diabetes	 55.4<br>15.1<br>28.3<br>1.2	 56.5<br>14.1<br>28.1<br>1.2	>0.05
Antihypertensive medication use (%)	41.5	37.7	0.03
Lipid-modifying medication use (%)	37.9	33.4	0.01
Glucose-lowering medication use (%)	23.6	22.7	>0.05
Total cholesterol-to-HDL cholesterol ratio	3.38 [2.76;4.17]	3.67 [3.00;4.55]	<0.01
Triglycerides (mmol/L)	1.22 [0.89;1.73]	1.18 [0.86;1.70]	>0.05
Dutch healthy diet index	83.70 (14.67)	81.99 (14.81)	<0.01
eGFR	89.03 [79.01;98.46]	90.25 [79.08;99.56]	>0.05
Albuminuria (%)	8.9	8.3	>0.05
Total physical activity (h/day)	1.96 [1.49;2.44]	1.92 [1.44;2.38]	>0.05

Light intensity physical activity (h/day)	5.41 (1.52)	5.43 (1.61)	>0.05
Moderate-to-vigorous intensity physical activity (h/day)	0.85 [0.58;1.16]	0.79 [0.50;1.06]	0.03
Vigorous intensity physical activity (h/day)	0.09 [0.04;0.19]	0.07 [0.03;0.17]	0.03
Sedentary time (h/day)	9.41 (1.68)	9.35 (1.77)	>0.05
Sedentary breaks (n/day)	37.42 (8.56)	38.32 (8.90)	>0.05
Prolonged sedentary bouts (n/day)	4.86 [3.71;5.86]	4.71 [3.71;5.67]	>0.05
Hs-cTnI	1.90 [1.20;3.00]	2.1 [1.30;3.20]	>0.05
Hs-cTnT	5.50[3.90;8.04]	5.33 [3.67;7.67]	>0.05
NT-proBNP	6.08 [3.32;10.90]	6.13 [3.74;10.73]	>0.05

Values are means (SD) or median [Q1-Q3], unless stated otherwise. BMI, body mass index; eGFR, estimated glomerular filtration rate; Hs-cTnI, high-sensitivity cardiac troponins I; Hs-cTnT, high-sensitivity cardiac troponins T; NT-proBNP, N-terminal pro-B-type natriuretic peptide.

Supplementary table 2. Associations of physical activity and sedentary behavior with Hs-cTnI (categorical) (n=2370)

	Total physical activity			
	Q1(n=592) (11.4-89.4 min/day)	Q2 (n=593) (89.4-117.0 min/day)	Q3 (n=593) (117.0-146.4 min/day)	Q4 (n=592) (146.4-322.8 min/day)
Model 1	Ref	**-0.16 (-0.26;-0.05)**	**-0.16 (-0.27;-0.06)**	-0.09 (-0.20;0.01)
Model 2	Ref	**-0.17 (-0.27;-0.06)**	**-0.17 (-0.28;-0.07)**	-0.10 (-0.21;0.01)
Model 3	Ref	-0.12 (-0.22;-0.01)	-0.09 (-0.19;0.02)	0.01 (-0.10;0.12)
	Light intensity physical activity			
	Q1 (n=592) (46.2-259.2 min/day)	Q2 (n=593) (259.2-319.8 min/day)	Q3 (n=593) (319.8-385.8 min/day)	Q4 (n=592) (385.8-734.4 min/day)
Model 1	Ref	**-0.21 (-0.31;-0.10)**	**-0.15 (-0.25;-0.04)**	**-0.11 (-0.22;-0.001)**
Model 2	Ref	**-0.21 (-0.31;-0.11)**	**-0.15 (-0.26;-0.04)**	-0.10 (-0.21;0.01)
Model 3	Ref	**-0.18 (-0.28;-0.08)**	**-0.10 (-0.21;-0.001)**	-0.03 (-0.13;0.08)
Model 4	Ref	**-0.19 (-0.29;-0.09)**	**-0.12 (-0.22;-0.01)**	-0.05 (-0.15;0.06)
	Moderate-to-vigorous intensity physical activity			
	Q1(n=592) (0.6-34.8 min/day)	Q2 (n=593) (34.8-51.0 min/day)	Q3 (n=593) (51.0-69.6 min/day)	Q4 (n=592) (69.6-244.2 min/day)
Model 1	Ref	-0.05 (-0.16;0.05)	-0.07 (-0.18;0.03)	-0.07 (-0.18;0.04)
Model 2	Ref	-0.06 (-0.17;0.04)	-0.09 (-0.20;0.02)	-0.09 (-0.19;0.03)
Model 3	Ref	-0.03 (-0.13;0.08)	-0.02 (-0.12;0.08)	0.04 (-0.07;0.15)
Model 4	Ref	-0.02 (-0.12;0.09)	-0.003 (-0.11;0.11)	0.06 (-0.05;0.18)
	Vigorous intensity physical activity			
	Q1(n=592) (0.0-2.4 min/day)	Q2 (n=593) (2.4-5.4 min/day)	Q3 (n=593) (5.4-11.4 min/day)	Q4 (n=592) (11.4-107.4 min/day)

Model 1	Ref	-0.01 (-0.11;0.10)	0.000 (-0.11;0.11)	0.11 (-0.01;0.21)
Model 2	Ref	-0.01 (-0.11;0.10)	-0.01 (-0.12;0.10)	0.10 (-0.01;0.21)
Model 3	Ref	0.03 (-0.08;0.13)	0.06 (-0.04;0.17)	**0.22 (0.11;0.33)**
Model 4	Ref	0.04 (-0.06;0.14)	0.08 (-0.03;0.18)	**0.24 (0.13;0.35)**
	Sedentary time			
	Q1 (n=592) (148.8-495.0 min/day)	Q2 (n=593) (495.0-564.6 min/day)	Q3 (n=593) (564.6-634.8 min/day)	Q4 (n=592) (634.8-952.8 min/day)
Model 1	Ref	-0.02 (-0.13;0.08)	-0.05 (-0.16;0.05)	**0.17 (0.06;0.28)**
Model 2	Ref	-0.03 (-0.14;0.07)	-0.06 (-0.17;0.05)	**0.16 (0.05;0.27)**
Model 3	Ref	-0.07 (-0.17;0.03)	**-0.12 (-0.22;-0.02)**	0.07 (-0.05;0.17)
Model 4	Ref	-0.05 (-0.15;0.05)	-0.09 (-0.19;0.02)	0.11 (-0.003;0.23)

Regression results are presented as unstandardized coefficients (β), with 95% confidence intervals [95% CI]. Boldface indicates statistical significance ($p<0.05$). Model 1 was adjusted for age, sex, glucose metabolism status. Model 2 was additionally adjusted for smoking, Dutch healthy diet index and level of education. Model 3 was additionally adjusted for history of cardiovascular disease, **waist circumference**, mobility limitation (yes/no), triglycerides, total cholesterol-to-HDL cholesterol ratio, use of lipid-modifying medication, use of anti-hypertensive medication, office systolic blood pressure, estimated glomerular filtration rate and albuminuria. For the sedentary behavior and Light intensity physical activity, model 4 was additionally adjusted for moderate-to-vigorous physical activity. For moderate-to-vigorous physical activity and vigorous physical activity, model 4 was additionally adjusted for sedentary time.

Supplementary table 3. Associations of physical activity and sedentary behavior with Hs-cTnT (categorical) (n=2370)

	Total physical activity			
	Q1(n=592) (11.4-89.4 min/day)	Q2 (n=593) (89.4-117.0 min/day)	Q3 (n=593) (117.0-146.4 min/day)	Q4 (n=592) (146.4-322.8 min/day)
Model 1	Ref	**-0.20 (-0.29;0.11)**	**-0.13 (-0.22;0.04)**	**-0.13 (-0.22;0.03)**
Model 2	Ref	**-0.19 (-0.28;-0.09)**	**-0.12 (-0.21;-0.03)**	**-0.11 (-0.21;-0.02)**
Model 3	Ref	**-0.11 (-0.20;-0.02)**	0.000 (-0.09;0.09)	0.03 (-0.07;0.12)
	Light intensity physical activity			
	Q1 (n=592) (46.2-259.2 min/day)	Q2 (n=593) (259.2-319.8 min/day)	Q3 (n=593) (319.8-385.8 min/day)	Q4 (n=592) (385.8-734.4 min/day)
Model 1	Ref	**-0.10 (-0.19;-0.01)**	-0.09 (-0.18;0.01)	0.06 (-0.04;0.15)
Model 2	Ref	-0.07 (-0.16;0.02)	-0.05 (-0.15;0.04)	**0.010 (0.01;0.20)**
Model 3	Ref	-0.06 (-0.14;0.03)	-0.01 (-0.10;0.08)	**0.16 (0.07;0.25)**
Model 4	Ref	-0.05 (-0.14;0.03)	-0.01 (-0.10;0.08)	**0.16 (0.07;0.26)**
	Moderate-to-vigorous intensity physical activity			
	Q1(n=592) (0.6-34.8 min/day)	Q2 (n=593) (34.8-51.0 min/day)	Q3 (n=593) (51.0-69.6 min/day)	Q4 (n=592) (69.6-244.2 min/day)
Model 1	Ref	**-0.12 (-0.21;-0.03)**	**-0.13 (-0.23;-0.04)**	**-0.15 (-0.24;-0.06)**
Model 2	Ref	**-0.12 (-0.21;-0.02)**	**-0.13 (-0.22;-0.03)**	**-0.14 (-0.24;-0.05)**
Model 3	Ref	-0.06 (-0.15;0.03)	-0.04 (-0.13;0.06)	-0.003 (-0.10;0.09)
Model 4	Ref	-0.07 (-0.16;0.02)	-0.05 (-0.15;0.04)	-0.03 (-0.13;0.07)
	Vigorous physical activity			
	Q1(n=592) (0.0-2.4 min/day)	Q2 (n=593) (2.4-5.4 min/day)	Q3 (n=593) (5.4-11.4 min/day)	Q4 (n=592) (11.4-107.4 min/day)
Model 1	Ref	0.01 (-0.09;0.10)	-0.08 (-0.17;0.01)	-0.04 (-0.14;0.05)

Model 2	Ref	0.02 (-0.08;0.11)	-0.07 (-0.17;0.02)	-0.03 (-0.13;0.07)
Model 3	Ref	0.06 (-0.03;0.15)	-0.01 (-0.10;0.09)	0.10 (0.01;0.20)
Model 4	Ref	0.05 (-0.04;0.14)	-0.02 (-0.11;0.08)	0.09 (-0.01;0.19)
	Sedentary time			
	Q1 (n=592) (148.8-495.0 min/day)	Q2 (n=593) (495.0-564.6 min/day)	Q3 (n=593) (564.6-634.8 min/day)	Q4 (n=592) (634.8-952.8 min/day)
Model 1	Ref	**-0.09 (-0.18;-0.001)**	**-0.10 (-0.19;-0.01)**	0.05 (-0.05;0.14)
Model 2	Ref	**-0.10 (-0.18;-0.01)**	**-0.11 (-0.20;-0.02)**	0.03 (-0.06;0.13)
Model 3	Ref	**-0.13 (-0.22;-0.05)**	**-0.18 (-0.26;-0.09)**	-0.08 (-0.17;0.02)
Model 4	Ref	**-0.13 (-0.22;-0.05)**	**-0.18 (-0.27;-0.09)**	-0.08 (-0.18;0.02)

Regression results are presented as unstandardized coefficients (β), with 95% confidence intervals [95% CI]. Boldface indicates statistical significance ($p<0.05$). Model 1 was adjusted for age, sex, glucose metabolism status. Model 2 was additionally adjusted for smoking, Dutch healthy diet index and level of education. Model 3 was additionally adjusted for history of cardiovascular disease, **waist circumference**, mobility limitation (yes/no), triglycerides, total cholesterol-to-HDL cholesterol ratio, use of lipid-modifying medication, use of anti-hypertensive medication, office systolic blood pressure, estimated glomerular filtration rate and albuminuria. For the sedentary behavior and Light intensity physical activity, model 4 was additionally adjusted for moderate-to-vigorous physical activity. For moderate-to-vigorous physical activity and vigorous physical activity, model 4 was additionally adjusted for sedentary time.

Supplementary table 4. Associations of physical activity and sedentary behavior with NT-proBNP (categorical) (n=2370)

	Total physical activity			
	Q1(n=592) (11.4-89.4 min/day)	Q2 (n=593) (89.4-117.0 min/day)	Q3 (n=593) (117.0-146.4 min/day)	Q4 (n=592) (146.4-322.8 min/day)
Model 1	Ref	-0.08 (-0.19;0.03)	**-0.12 (-0.23;-0.01)**	**-0.16 (-0.27;-0.05)**
Model 2	Ref	-0.07 (-0.18;0.04)	-0.10 (-0.21;0.01)	**-0.14 (-0.25;-0.02)**
Model 3	Ref	-0.06 (-0.16;0.05)	-0.05 (-0.15;0.06)	-0.09 (-0.20;0.02)
	Light intensity physical activity			
	Q1 (n=592) (46.2-259.2 min/day)	Q2 (n=593) (259.2-319.8 min/day)	Q3 (n=593) (319.8-385.8 min/day)	Q4 (n=592) (385.8-734.4 min/day)
Model 1	Ref	-0.01 (-0.12;0.10)	-0.02 (-0.13;0.10)	-0.03 (-0.15;0.08)
Model 2	Ref	-0.004 (-0.11;0.11)	-0.001 (-0.11;0.11)	-0.02 (-0.14;0.09)
Model 3	Ref	0.004 (-0.10;0.11)	0.02 (-0.09;0.13)	-0.002 (-0.11;0.11)
Model 4	Ref	0.02 (-0.09;0.12)	0.03 (-0.07;0.14)	0.02 (-0.09;0.13)
	Moderate-to-vigorous intensity physical activity			
	Q1(n=592) (0.6-34.8 min/day)	Q2 (n=593) (34.8-51.0 min/day)	Q3 (n=593) (51.0-69.6 min/day)	Q4 (n=592) (69.6-244.2 min/day)
Model 1	Ref	-0.02 (-0.13;0.09)	-0.03 (-0.14;0.08)	**-0.17 (-0.28;-0.05)**
Model 2	Ref	-0.01 (-0.12;0.10)	-0.02 (-0.13;0.09)	**-0.14 (-0.26;-0.03)**
Model 3	Ref	0.001 (-0.11;0.11)	0.02 (-0.09;0.13)	-0.09 (-0.20;0.02)
Model 4	Ref	-0.001 (-0.11;0.11)	0.02 (-0.09;0.13)	-0.09 (-0.21;0.03)
	Vigorous intensity physical activity			
	Q1(n=592) (0.0-2.4 min/day)	Q2 (n=593) (2.4-5.4 min/day)	Q3 (n=593) (5.4-11.4 min/day)	Q4 (n=592) (11.4-107.4 min/day)
Model 1	Ref	-0.07 (-0.17;0.04)	-0.06 (-0.17;0.06)	**-0.19 (-0.30;-0.08)**

Model 2	Ref	-0.05 (-0.16;0.06)	-0.05 (-0.16;0.06)	**-0.17 (-0.29;-0.06)**
Model 3	Ref	-0.04 (-0.14;0.07)	-0.04 (-0.15;0.07)	**-0.13 (-0.25;-0.02)**
Model 4	Ref	-0.04 (-0.15;0.07)	-0.04 (-0.15;0.07)	**-0.13 (-0.25;-0.02)**
	Sedentary time			
	Q1 (n=592) (148.8-495.0 min/day)	Q2 (n=593) (495.0-564.6 min/day)	Q3 (n=593) (564.6-634.8 min/day)	Q4 (n=592) (634.8-952.8 min/day)
Model 1	Ref	0.08 (-0.03;0.19)	-0.02 (-0.13;0.09)	0.07 (-0.05;0.18)
Model 2	Ref	0.08 (-0.03;0.19)	-0.03 (-0.14;0.08)	0.05 (-0.06;0.17)
Model 3	Ref	0.07 (-0.04;0.17)	-0.04 (-0.15;0.06)	0.03 (-0.09;0.14)
Model 4	Ref	0.05 (-0.05;0.15)	-0.07 (-0.18;0.04)	-0.02 (-0.14;0.10)

Regression results are presented as unstandardized coefficients (β), with 95% confidence intervals [95% CI]. Boldface indicates statistical significance ($p<0.05$). Model 1 was adjusted for age, sex, glucose metabolism status. Model 2 was additionally adjusted for smoking, Dutch healthy diet index and level of education. Model 3 was additionally adjusted for history of cardiovascular disease, **waist circumference**, mobility limitation (yes/no), triglycerides, total cholesterol-to-HDL cholesterol ratio, use of lipid-modifying medication, use of anti-hypertensive medication, office systolic blood pressure, estimated glomerular filtration rate and albuminuria. For the sedentary behavior and Light intensity physical activity, model 4 was additionally adjusted for moderate-to-vigorous physical activity. For moderate-to-vigorous physical activity and vigorous physical activity, model 4 was additionally adjusted for sedentary time.

Supplementary table 5. Associations of physical activity and sedentary behavior with Hs-cTnI (categorical) (n=2370)

	Total physical activity			
	Q1(n=592) (11.4-89.4 min/day)	Q2 (n=593) (89.4-117.0 min/day)	Q3 (n=593) (117.0-146.4 min/day)	Q4 (n=592) (146.4-322.8 min/day)
Model 1	Ref	**-0.15 (-0.27;-0.04)**	**-0.16 (-0.27;-0.04)**	-0.09 (-0.20;0.03)
Model 2	Ref	**-0.16 (-0.28;-0.05)**	**-0.17 (-0.29;-0.05)**	-0.10 (-0.22;0.02)
Model 3	Ref	**-0.11 (-0.22;-0.01)**	-0.08 (-0.20;0.03)	0.01 (-0.11;0.12)
	Light intensity physical activity			
	Q1 (n=592) (46.2-259.2 min/day)	Q2 (n=593) (259.2-319.8 min/day)	Q3 (n=593) (319.8-385.8 min/day)	Q4 (n=592) (385.8-734.4 min/day)
Model 1	Ref	**-0.20 (-0.32;-0.09)**	**-0.12 (-0.24;-0.01)**	-0.12 (-0.24;0.004)
Model 2	Ref	**-0.21 (-0.32;-0.09)**	**-0.13 (-0.25;-0.01)**	-0.11 (-0.23;0.01)
Model 3	Ref	**-0.19 (-0.30;-0.08)**	-0.10 (-0.21;0.01)	-0.05 (-0.17;0.07)
Model 4	Ref	**-0.20 (-0.31;-0.09)**	-0.11 (-0.23;0.000)	-0.07 (-0.19;0.05)
	Moderate-to-vigorous intensity physical activity			
	Q1(n=592) (0.6-34.8 min/day)	Q2 (n=593) (34.8-51.0 min/day)	Q3 (n=593) (51.0-69.6 min/day)	Q4 (n=592) (69.6-244.2 min/day)
Model 1	Ref	-0.03 (-0.14;0.09)	-0.09 (-0.21;0.03)	-0.05 (-0.17;0.07)
Model 2	Ref	-0.04 (-0.16;0.08)	-0.11 (-0.23;0.01)	-0.07 (-0.23;0.01)
Model 3	Ref	0.02 (-0.09;0.13)	-0.04 (-0.15;0.08)	0.05 (-0.07;0.17)
Model 4	Ref	0.03 (-0.09;0.14)	-0.02 (-0.14;0.10)	0.07 (-0.05;0.20)
	Vigorous intensity physical activity			
	Q1(n=592) (0.0-2.4 min/day)	Q2 (n=593) (2.4-5.4 min/day)	Q3 (n=593) (5.4-11.4 min/day)	Q4 (n=592) (11.4-107.4 min/day)
Model 1	Ref	0.01 (-0.11;0.12)	0.01 (-0.11;0.13)	0.11 (-0.02;0.23)
Model 2	Ref	0.000 (-0.12;0.12)	-0.001 (-0.12;0.12)	0.09 (-0.03;0.22)
Model 3	Ref	0.03 (-0.09;0.14)	0.07 (-0.04;0.19)	**0.21 (0.09;0.33)**
Model 4	Ref	0.04 (-0.08;0.15)	0.09 (-0.03;0.21)	**0.22 (0.10;0.35)**
	Sedentary time			
	Q1 (n=592) (148.8-495.0 min/day)	Q2 (n=593) (495.0-564.6 min/day)	Q3 (n=593) (564.6-634.8 min/day)	Q4 (n=592) (634.8-952.8 min/day)
Model 1	Ref	-0.02 (-0.13;0.08)	-0.05 (-0.16;0.05)	**0.17 (0.06;0.28)**

Model 2	Ref	-0.03 (-0.14;0.07)	-0.06 (-0.17;0.05)	**0.16 (0.05;0.27)**
Model 3	Ref	-0.07 (-0.17;0.03)	**-0.12 (-0.22;-0.02)**	0.06 (-0.05;0.17)
Model 4	Ref	-0.05 (-0.15;0.05)	-0.09 (-0.19;0.02)	0.11 (-0.002;0.23)

Regression coefficients (β) represents β the increase/decrease in cardiac biomarker for every standard deviation (SD) increase in physical activity/sedentary behavior. Boldface indicates statistical significance ($p<0.05$). Model 1 was adjusted for age, sex, glucose metabolism status. Model 2 was additionally adjusted for smoking, Dutch healthy diet index, level of education and ECG abnormalities. Model 3 was additionally adjusted for history of cardiovascular disease, BMI, mobility limitation (yes/no), triglycerides, total cholesterol-to-HDL cholesterol ratio, use of lipid-modifying medication, use of anti-hypertensive medication, **24-hour systolic blood pressure**, estimated glomerular filtration rate and albuminuria. For the sedentary behavior and Light intensity physical activity, model 4 was additionally adjusted for moderate-to-vigorous physical activity. For moderate-to-vigorous physical activity and vigorous physical activity, model 4 was additionally adjusted for light intensity physical activity.

Supplementary table 6. Associations of physical activity and sedentary behavior with Hs-cTnT (categorical) (n=2370)

	Total physical activity			
	Q1(n=592) (11.4-89.4 min/day)	Q2 (n=593) (89.4-117.0 min/day)	Q3 (n=593) (117.0-146.4 min/day)	Q4 (n=592) (146.4-322.8 min/day)
Model 1	Ref	**-0.19 (-0.29;-0.09)**	**-0.11 (-0.21;-0.01)**	**-0.12 (-0.22;-0.02)**
Model 2	Ref	**-0.17 (-0.27;-0.07)**	-0.10 (-0.20;0.01)	**-0.11 (-0.21;-0.01)**
Model 3	Ref	**-0.11 (-0.20;-0.01)**	0.01 (-0.09;0.11)	0.01 (-0.09;0.11)
	Light intensity physical activity			
	Q1 (n=592) (46.2-259.2 min/day)	Q2 (n=593) (259.2-319.8 min/day)	Q3 (n=593) (319.8-385.8 min/day)	Q4 (n=592) (385.8-734.4 min/day)
Model 1	Ref	-0.09 (-0.19;0.01)	-0.09 (-0.19;0.01)	0.07 (-0.03;0.17)
Model 2	Ref	-0.07 (-0.17;0.03)	-0.06 (-0.17;0.04)	**0.12 (0.01;0.22)**
Model 3	Ref	-0.07 (-0.16;0.03)	-0.03 (-0.13;0.06)	**0.15 (0.06;0.25)**
Model 4	Ref	-0.07 (-0.16;0.03)	-0.03 (-0.13;0.06)	**0.16 (0.06;0.26)**
	Moderate-to-vigorous intensity physical activity			
	Q1(n=592) (0.6-34.8 min/day)	Q2 (n=593) (34.8-51.0 min/day)	Q3 (n=593) (51.0-69.6 min/day)	Q4 (n=592) (69.6-244.2 min/day)
Model 1	Ref	**-0.11 (-0.21;-0.01)**	**-0.12 (-0.22;-0.02)**	**-0.14 (-0.25;-0.04)**
Model 2	Ref	-0.10 (-0.20;0.003)	**-0.11 (-0.22;-0.01)**	**-0.14 (-0.24;-0.04)**
Model 3	Ref	-0.04 (-0.14;0.05)	-0.04 (-0.13;0.06)	-0.01 (-0.11;0.09)
Model 4	Ref	-0.05 (-0.15;0.05)	-0.05 (-0.15;0.05)	-0.03 (-0.14;0.07)
	Vigorous intensity physical activity			
	Q1(n=592) (0.0-2.4 min/day)	Q2 (n=593) (2.4-5.4 min/day)	Q3 (n=593) (5.4-11.4 min/day)	Q4 (n=592) (11.4-107.4 min/day)
Model 1	Ref	0.05 (-0.05;0.15)	-0.06 (-0.17;0.04)	-0.02 (-0.13;0.08)

Model 2	Ref	0.06 (-0.04;0.16)	-0.06 (-0.16;0.05)	-0.01 (-0.12;0.09)
Model 3	Ref	0.08 (-0.01;0.18)	0.01 (-0.09;0.11)	0.10 (-0.004;0.20)
Model 4	Ref	0.08 (-0.02;0.17)	0.001 (-0.10;0.10)	0.09 (-0.01;0.19)
	Sedentary time			
	Q1 (n=592) (148.8-495.0 min/day)	Q2 (n=593) (495.0-564.6 min/day)	Q3 (n=593) (564.6-634.8 min/day)	Q4 (n=592) (634.8-952.8 min/day)
Model 1	Ref	-0.09 (-0.18;0.01)	**-0.10 (-0.20;-0.004)**	0.05 (-0.06;0.15)
Model 2	Ref	-0.09 (-0.19;0.01)	**-0.11 (-0.21;-0.01)**	0.03 (-0.08;0.14)
Model 3	Ref	**-0.12 (-0.22;-0.03)**	**-0.16 (-0.26;-0.07)**	-0.07 (-0.17;0.03)
Model 4	Ref	**-0.13 (-0.22;-0.03)**	**-0.16 (-0.26;-0.07)**	-0.07 (-0.18;0.04)

Regression coefficients (β) represents β the increase/decrease in cardiac biomarker for every standard deviation (SD) increase in physical activity/sedentary behavior. Boldface indicates statistical significance ($p<0.05$). Model 1 was adjusted for age, sex, glucose metabolism status. Model 2 was additionally adjusted for smoking, Dutch healthy diet index, level of education and ECG abnormalities. Model 3 was additionally adjusted for history of cardiovascular disease, BMI, mobility limitation (yes/no), triglycerides, total cholesterol-to-HDL cholesterol ratio, use of lipid-modifying medication, use of anti-hypertensive medication, **24-hour systolic blood pressure**, estimated glomerular filtration rate and albuminuria. For the sedentary behavior and Light intensity physical activity, model 4 was additionally adjusted for moderate-to-vigorous physical activity. For moderate-to-vigorous physical activity and vigorous physical activity, model 4 was additionally adjusted for light intensity physical activity.

Supplementary table 7. Associations of physical activity and sedentary behavior with NT-proBNP (categorical) (n=2370)

	Total physical activity			
	Q1(n=592) (11.4-89.4 min/day)	Q2 (n=593) (89.4-117.0 min/day)	Q3 (n=593) (117.0-146.4 min/day)	Q4 (n=592) (146.4-322.8 min/day)
Model 1	Ref	-0.08 (-0.19;0.04)	-0.11 (-0.22;0.01)	**-0.17 (-0.28;-0.05)**
Model 2	Ref	-0.07 (-0.18;0.05)	-0.09 (-0.21;0.03)	**-0.14 (-0.26;-0.02)**
Model 3	Ref	-0.05 (-0.16;0.07)	-0.04 (-0.16;0.08)	-0.10 (-0.22;0.02)
	Light intensity physical activity			
	Q1 (n=592) (46.2-259.2 min/day)	Q2 (n=593) (259.2-319.8 min/day)	Q3 (n=593) (319.8-385.8 min/day)	Q4 (n=592) (385.8-734.4 min/day)
Model 1	Ref	0.01 (-0.11;0.13)	0.01 (-0.11;0.13)	-0.04 (-0.16;0.09)
Model 2	Ref	0.02 (-0.10;0.14)	0.02 (-0.10;0.14)	-0.03 (-0.15;0.10)
Model 3	Ref	0.02 (-0.10;0.13)	0.04 (-0.08;0.15)	-0.01 (-0.13;0.11)
Model 4	Ref	0.03 (-0.09;0.14)	0.05 (-0.07;0.16)	0.01 (-0.11;0.13)
	Moderate-to-vigorous intensity physical activity			
	Q1(n=592) (0.6-34.8 min/day)	Q2 (n=593) (34.8-51.0 min/day)	Q3 (n=593) (51.0-69.6 min/day)	Q4 (n=592) (69.6-244.2 min/day)
Model 1	Ref	0.01 (-0.11;0.13)	-0.03 (-0.15;0.09)	**-0.15 (-0.27;-0.03)**
Model 2	Ref	0.02 (-0.10;0.13)	-0.02 (-0.14;0.10)	**-0.13 (-0.25;-0.01)**
Model 3	Ref	0.04 (-0.08;0.16)	0.03 (-0.09;0.15)	-0.07 (-0.19;0.05)
Model 4	Ref	0.04 (-0.08;0.15)	0.02 (-0.10;0.14)	-0.08 (-0.21;0.05)
	Vigorous intensity physical activity			
	Q1(n=592) (0.0-2.4 min/day)	Q2 (n=593) (2.4-5.4 min/day)	Q3 (n=593) (5.4-11.4 min/day)	Q4 (n=592) (11.4-107.4 min/day)
Model 1	Ref	-0.04 (-0.15;0.08)	-0.06 (-0.18;0.06)	**-0.17 (-0.29;-0.05)**
Model 2	Ref	-0.03 (-0.15;0.09)	-0.06 (-0.18;0.07)	**-0.15 (-0.28;-0.03)**
Model 3	Ref	-0.02 (-0.13;0.10)	-0.04 (-0.15;0.08)	-0.10 (-0.22;0.02)
Model 4	Ref	-0.02 (-0.14;0.09)	-0.04 (-0.16;0.08)	-0.11 (-0.23;0.02)
	Sedentary time			
	Q1 (n=592) (148.8-495.0 min/day)	Q2 (n=593) (495.0-564.6 min/day)	Q3 (n=593) (564.6-634.8 min/day)	Q4 (n=592) (634.8-952.8 min/day)
Model 1	Ref	0.08 (-0.04;0.19)	-0.02 (-0.14;0.10)	0.05 (-0.07;0.17)

Model 2	Ref	0.07 (-0.05;0.19)	-0.03 (-0.15;0.09)	0.03 (-0.09;0.15)
Model 3	Ref	0.06 (-0.05;0.17)	-0.04 (-0.16;0.07)	0.000 (-0.12;0.12)
Model 4	Ref	0.05 (-0.07;0.16)	-0.07 (-0.19;0.05)	-0.04 (-0.17;0.09)

Regression coefficients (β) represents β the increase/decrease in cardiac biomarker for every standard deviation (SD) increase in physical activity/sedentary behavior. Boldface indicates statistical significance ($p<0.05$). Model 1 was adjusted for age, sex, glucose metabolism status. Model 2 was additionally adjusted for smoking, Dutch healthy diet index, level of education and ECG abnormalities. Model 3 was additionally adjusted for history of cardiovascular disease, BMI, mobility limitation (yes/no), triglycerides, total cholesterol-to-HDL cholesterol ratio, use of lipid-modifying medication, use of anti-hypertensive medication, **24-hour systolic blood pressure**, estimated glomerular filtration rate and albuminuria. For the sedentary behavior and Light intensity physical activity, model 4 was additionally adjusted for moderate-to-vigorous physical activity. For moderate-to-vigorous physical activity and vigorous physical activity, model 4 was additionally adjusted for light intensity physical activity.
